# Cardiovascular/anti‐inflammatory drugs repurposed for treating or preventing cancer: A systematic review and meta‐analysis of randomized trials

**DOI:** 10.1002/cam4.7049

**Published:** 2024-03-16

**Authors:** David J. Benjamin, Alyson Haslam, Vinay Prasad

**Affiliations:** ^1^ Hoag Family Cancer Institute Newport Beach California USA; ^2^ Department of Epidemiology and Biostatistics University of California San Francisco California United States

**Keywords:** cardiovascular drugs, drug repurposing, oncology trials, randomized trials

## Abstract

**Background:**

Due to encouraging pre‐clinical data and supportive observational studies, there has been growing interest in applying cardiovascular drugs (including aspirin, angiotensin‐converting enzyme [ACE] inhibitors, statins, and metformin) approved to treat diseases such as hypertension, hyperlipidemia, and diabetes mellitus to the field of oncology. Moreover, given growing costs with cancer care, these medications have offered a potentially more affordable avenue to treat or prevent recurrence of cancer.

We sought to investigate the anti‐cancer effects of drugs repurposed from cardiology or anti‐inflammatories to treat cancer. We specifically evaluated the following drug classes: HMG‐CoA reductase inhibitors (statins), cyclo‐oxygenase inhibitors, aspirin, metformin, and both angiotensin receptor blockers (ARBs) and angiotensin‐converting enzyme inhibitors. We also included non‐steroidal anti‐inflammatory drugs (NSAIDs) because they exert a similar mechanism to aspirin by blocking prostaglandins and reducing inflammation that is thought to promote the development of cancer.

**Methods:**

We performed a systematic literature review using PubMed and Web of Science with search terms including “aspirin,” “NSAID,” “statin” (including specific statin drug names), “metformin,” “ACE inhibitors,” and “ARBs” (including specific anti‐hypertensive drug names) in combination with “cancer.” Searches were limited to human studies published between 2000 and 2023.

**Main Outcomes and Measures:**

The number and percentage of studies reported positive results and pooled estimates of overall survival, progression‐free survival, response, and disease‐free survival.

**Results:**

We reviewed 3094 titles and included 67 randomized clinical trials. The most common drugs that were tested were metformin (*n* = 21; 30.9%), celecoxib (*n* = 20; 29.4%), and simvastatin (*n* = 8; 11.8%). There was only one study that tested cardiac glycosides and none that studied ACE inhibitors. The most common tumor types were non‐small‐cell lung cancer (*n* = 19; 27.9%); breast (*n* = 8; 20.6%), colorectal (*n* = 7; 10.3%), and hepatocellular (*n* = 6; 8.8%). Most studies were conducted in a phase II trial (*n* = 38; 55.9%). Most studies were tested in metastatic cancers (*n* = 49; 72.1%) and in the first‐line setting (*n* = 36; 521.9%). Four studies (5.9%) were stopped early because of difficulty with accrual. The majority of studies did not demonstrate an improvement in either progression‐free survival (86.1% of studies testing progression‐free survival) or in overall survival (94.3% of studies testing overall survival). Progression‐free survival was improved in five studies (7.4%), and overall survival was improved in three studies (4.4%). Overall survival was significantly worse in two studies (3.8% of studies testing overall survival), and progression‐free survival was worse in one study (2.8% of studies testing progression‐free survival).

**Conclusions and Relevance:**

Despite promising pre‐clinical and population‐based data, cardiovascular drugs and anti‐inflammatory medications have overall not demonstrated benefit in the treatment or preventing recurrence of cancer. These findings may help guide future potential clinical trials involving these medications when applied in oncology.

## 1 INTRODUCTION

Cancer remains the leading cause of morbidity in many countries with an estimated 19.3 million new cancer cases and nearly 10.0 million deaths globally in 2020 alone.^1^ Despite the development and approval of therapeutics such as immune checkpoint inhibitors and drugs targeting specific oncogenic driver mutations, many individuals with cancer remain ineligible for these therapies and many cancers remain incurable.^2^, ^3^, ^4^ As such, cancer continues to inflict devastating financial and societal consequences worldwide.^5^ Given the significant time and financial costs associated with developing new cancer drugs, there has historically been interest in repurposing drugs approved in other settings to the treatment or prevention of several types of cancer.^6^ Repurposed drugs carry the potential to fill a void in drug development.

Repurposed drugs offer several potential benefits. Generally, much is known about the safety profile of each repurposed drug owing to each drug's wide‐scale use. Second, many of these drugs are widely available and affordable. If repurposed drugs were effective in improving cancer outcomes, they would be a boon to the global oncology community. There are several research groups, such as the Repurposing Drugs in Oncology (ReDO) Project, who are avidly investigating the use of cardiovascular drugs in treating or preventing cancer.^7^

Due to encouraging pre‐clinical data and supportive observational studies, there has been growing interest in applying cardiovascular drugs (including aspirin, angiotensin‐converting enzyme [ACE] inhibitors, statins, and metformin), approved to treat diseases such as hypertension, hyperlipidemia, and diabetes mellitus, to the field of oncology.^8^ For example, data suggest that statins such as lovastatin may lead to tumor‐specific apoptosis in the setting of acute myeloid leukemia.^9^ In addition, data suggest that metformin can activate the adenosine monophosphate‐activated kinase pathway through interactions with p53, and thereby prevent cancer cell proliferation.^10^ Moreover, pre‐clinical studies have demonstrated that aspirin can reduce colon cancer cell growth in a xenograft model and lead to downregulation of specificity protein transcription factors that play a role in promoting oncogenes associated with tumor progression and metastasis.^11^ Several other pre‐clinical studies have demonstrated similar results with commonly used cardiovascular drugs in the setting of several cancer types and have consequently generated enthusiasm for these drugs.

Observational data also support these aforementioned claims. Metformin, statins, aspirin, ACE inhibitors, and beta blockers have all demonstrated improved survival among individuals with cancer compared to those who do not receive these medications.^11^, ^12^, ^13^, ^14^, ^15^ Favorable evidence for repurposed drugs from observational research is present both in adjuvant and metastatic settings.^11^, ^16^

At the same time, repurposed drugs rarely demonstrate RECIST (Response Evaluation Criteria in Solid Tumors) 1.1 tumor responses (30%) in studies, an important heuristic for effective cancer drugs.^17^, ^18^ While multiple observational and population‐based studies have suggested survival benefit or reduction in cancer recurrence with these cardiovascular drugs, several recently published randomized controlled trials have not confirmed these advantages.^19^

As the costs of cancer drugs continue to rise, repurposing of inexpensive cardiovascular drugs in oncology offers a promising and potentially cost‐effective avenue to cancer prevention and treatment. We sought to take further evaluate this approach. We undertook a systematic review and meta‐analysis to evaluate the effectiveness of repurposing cardiovascular drugs in oncology as studied in randomized controlled trials.

## 2 METHODS

We sought to investigate the anti‐cancer effects of drugs repurposed from cardiology or anti‐inflammatories to treat cancer. We specifically evaluated the following drug classes: HMG‐CoA reductase inhibitors (statins), cyclo‐oxygenase inhibitors, aspirin, metformin, and both angiotensin receptor blockers and angiotensin‐converting enzyme inhibitors. We also included non‐steroidal anti‐inflammatory drugs (NSAIDs) because they exert a similar mechanism to aspirin by blocking prostaglandins and reducing inflammation that is thought to promote the development of cancer.^20^

### 2.1 Search strategy

We performed a systematic literature review using PubMed and Web of Science with search terms including “aspirin,” “NSAID,” “statin” (including specific statin drug names), “metformin,” “ACE inhibitors,” and “ARBs” (including specific anti‐hypertensive drug names) in combination with “cancer.” For a full list of searches, please see supplemental methods. Searches were made on November 30, 2023.

### 2.2 Inclusion and exclusion criteria

We followed PRISMA guidelines throughout the study. Searches were limited to human studies published between 2000 and 2023. The review included all tumor types. Relevant studies were identified by two authors (D.J.B. and A.H.). We included studies if the study population comprised patients with cancer, if the study was a randomized trial, and if the study endpoint was survival, disease progression, response, or reduction in cancer recurrence. Studies could include any setting—metastatic, adjuvant, or neoadjuvant, as long as it was being used for anti‐tumor purposes. Studies needed to report on markers of overall survival and/or markers of response (e.g., response rate, disease‐free survival, and progression‐free survival). Studies were excluded for the following reasons: The study design was a meta‐analysis, review article, case–control study, or observational; the study endpoint was a biomarker change; reported on an outcome other than survival/cancer recurrence; included non‐cancerous conditions such as hyperplasia, adenomas, or in situ; was a secondary analysis (given the possibility for duplicate reporting of a study); tested a drug to prevent chemotherapy‐induced side‐effects (e.g., cardiotoxicity or hearing loss); tested the intervention as a chemoprevention strategy; or it was a trial protocol.

From each study, we abstracted the following information: the drug used in the intervention arm, the control arm, primary endpoint, tumor type, stage, line of therapy, the number of patients total, in the intervention arm, and in the control arm, the outcome, year of publication, phase, whether the trial was stopped early, and whether the trial demonstrated significant benefit or harm.

If more than two arms were tested, we used the comparisons between systemic therapy alone vs systemic therapy plus the cardiovascular drug. If intention‐to‐treat and per protocol results were both presented, we opted to use the intention‐to‐treat results.

### 2.3 Statistical analysis

Data for meta‐analysis were collected and analyzed using Excel, version 16.2 (Microsoft Corporation) and R statistical Software, version 4.2.1. We calculated study‐level descriptive statistics. For each main outcome (overall survival, progression‐free survival, recurrence, response rate, and disease‐free‐survival), we used the meta package of R to calculate pooled effect sizes, stratified by drug type (metformin, statins, aspirin, and other NSAIDs). We used a random effects model using the restricted maximum‐likelihood estimator with Knapp‐Hartung adjustments. We were initially unsure whether there would be notable heterogeneity in study findings, so we used a random effects model for calculating pooled estimates. Also, because there were multiple interventions assessed, we calculated pooled effects overall and by therapy subtype. Differences in subgroups were assessed using a *Q*‐test.

Because our study involved publicly available data and did not involve individual patient data, this study was not submitted for institutional review board, in accordance with 45 CFR §46.102(f). This report followed the Strengthening the Reporting of Observational Studies in Epidemiology reporting guidelines.

## 3 RESULTS

We reviewed 3094 titles and included 67 randomized clinical trials.^21^, ^22^, ^23^, ^24^, ^25^, ^26^, ^27^, ^28^, ^29^, ^30^, ^31^, ^32^, ^33^, ^34^, ^35^, ^36^, ^37^, ^38^, ^39^, ^40^, ^41^, ^42^, ^43^, ^44^, ^45^, ^46^, ^47^, ^48^, ^49^, ^50^, ^51^, ^52^, ^53^, ^54^, ^55^, ^56^, ^57^, ^58^, ^59^, ^60^, ^61^, ^62^, ^63^, ^64^, ^65^, ^66^, ^67^, ^68^, ^69^, ^70^, ^71^, ^72^, ^73^, ^74^, ^75^, ^76^, ^77^, ^78^, ^79^, ^80^, ^81^, ^82^, ^83^, ^84^, ^85^, ^86^, ^87^ The Supplemental Figure shows the process of selecting studies.

### 3.1 Study characteristics

**FIGURE 1:**
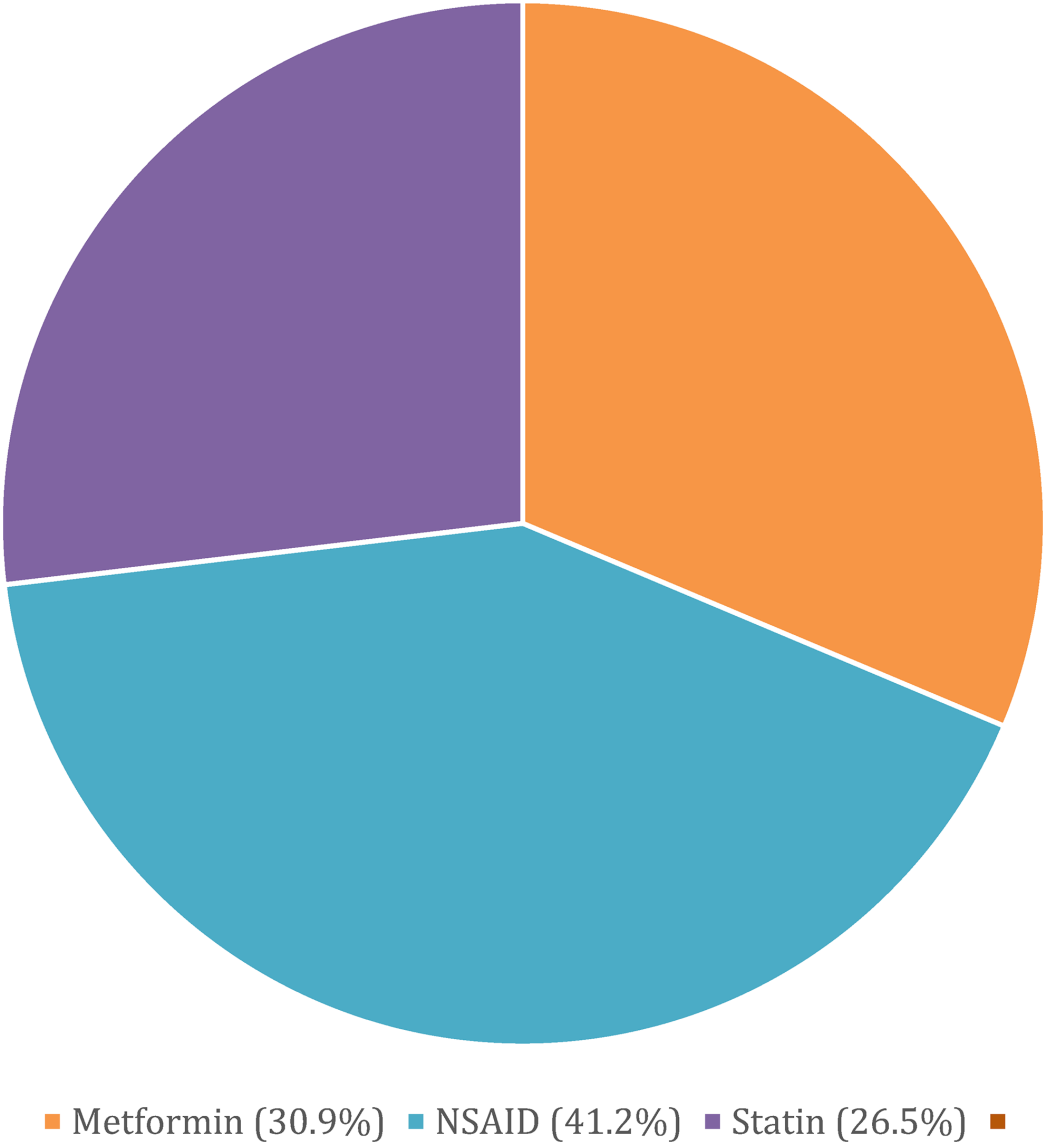
Studies (%) by drug class.

**FIGURE 2:**
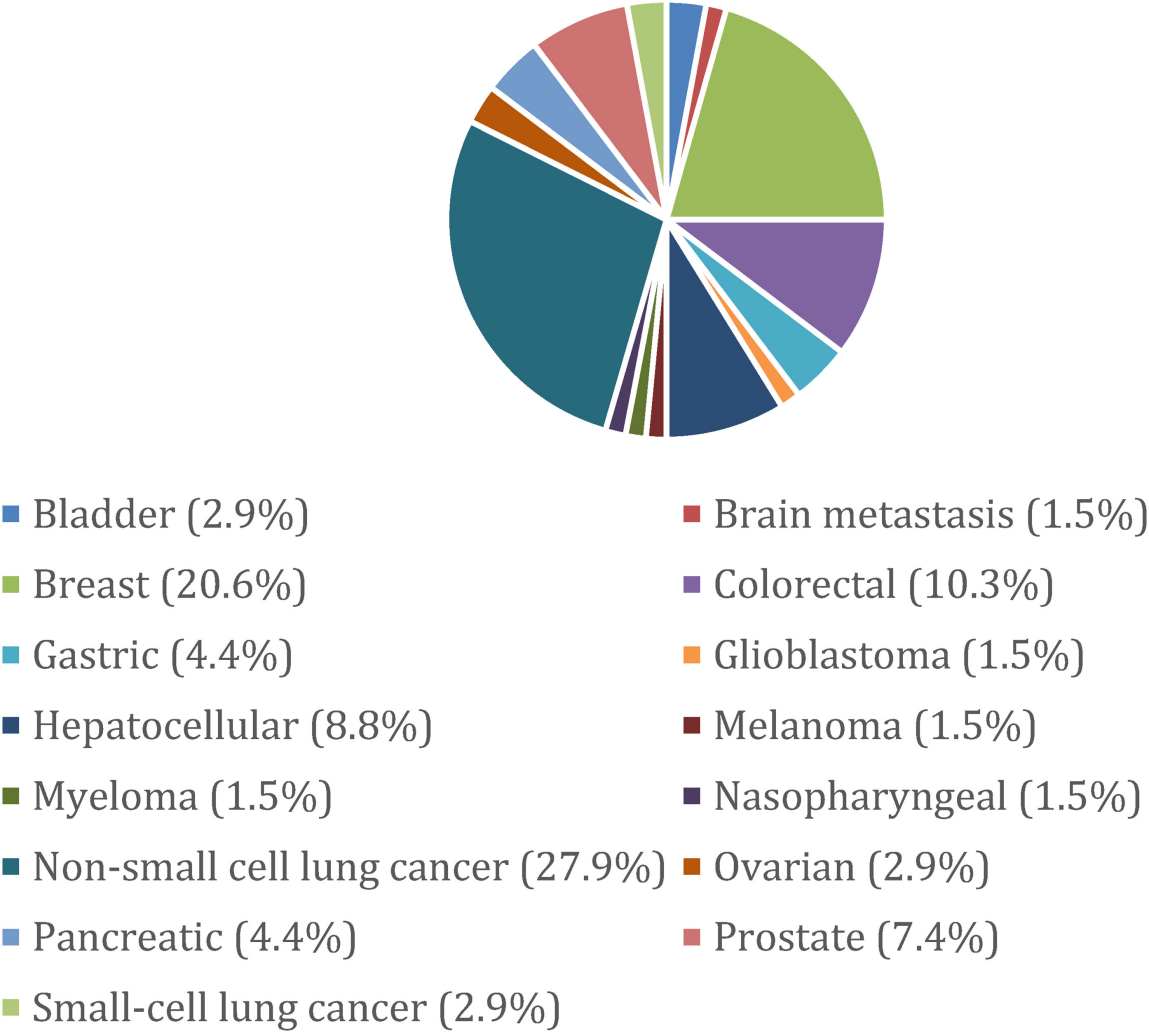
Studies (%) by tumor.

The median number of study participants was 120 (IQR: 70, 241; Table 1). The most common drugs that were tested were metformin (*n* = 21; 30.9%), celecoxib (*n* = 20; 29.4%), and simvastatin (*n* = 8; 11.8%). There was only one study that tested cardiac glycosides and none that studied ACE inhibitors. The most common tumor types were non‐small‐cell lung cancer (*n* = 19; 27.9%); breast (*n* = 14; 20.6%), colorectal (*n* = 7; 10.3%), and hepatocellular (*n* = 6; 8.8%). Most studies were conducted in a phase II trial (*n* = 38; 55.9%). Most studies were tested in metastatic cancers (*n* = 49; 72.1%) and in the first‐line setting (*n* = 36; 52.9%). Four studies (5.9%) were stopped early because of difficulty with accrual. Study characteristics are shown in Figures 1 and 2.

**TABLE 1:** Characteristics of randomized clinical trials investigating cardiovascular drugs as anti‐tumor therapies (*N* = 67).

Characteristic	Number of studies (%), unless otherwise indicated
Number of participants, median (IQR)	120 (70, 241)
Drug class	
Metformin	21 (30.9)
NSAID	28 (41.2)
Apricoxib	2 (2.9)
Aspirin	1 (1.5)
Celecoxib	20 (29.4)
Rofecoxib	3 (4.4)
Celecoxib + aspirin	1 (1.5)
Mefenamic acid	1 (1.5)
Statin	18 (26.5)
Atorvastatin	1 (1.5)
Pravastatin	7 (10.3)
Simvastatin	8 (11.8)
Lovastatin	2 (2.9)
Beta blocker + NSAID	1 (1.5)
Propranolol + etodolac	1 (1.5)
Tumor	
Bladder	2 (2.9)
Brain metastasis	1 (1.5)
Breast	14 (20.6)
Colorectal	7 (10.3)
Gastric	3 (4.4)
Glioblastoma	1 (1.5)
Hepatocellular	6 (8.8)
Melanoma	1 (1.5)
Myeloma	1 (1.5)
Nasopharyngeal	1 (1.5)
Non‐small‐cell lung cancer	19 (27.9)
Ovarian	2 (2.9)
Pancreatic	3 (4.4)
Prostate	5 (7.4)
Small‐cell lung cancer	2 (2.9)
Phase	
II	38 (55.9)
II–III	1 (1.5)
III	22 (32.4)
Not indicated	4 (5.9)
Pilot	3 (4.4)
Stopped early, yes	4 (5.9)
Demonstrated benefit, yes	
Disease‐free survival (*n* = 12)	0
Event‐free survival (*n* = 1)	0
Failure‐free survival (*n* = 1)	0
Overall survival (*n* = 53)	3 (4.4)
Progression‐free survival (*n* = 36)	5 (7.4)
Response (*n* = 32)	4 (5.9)
Time to progression (*n* = 4)	0
Setting	
Adjuvant	11 (16.2)
Any	7 (10.3)
First‐line	36 (52.9)
First/second	1 (1.5)
Neoadjuvant	3 (4.4)
Subsequent line	10 (14.7)

Abbreviation: NSAID, non‐steroidal anti‐inflammatory drugs.

### 3.2 Study results

The majority of studies did not demonstrate an improvement in either progression‐free survival (86.1% of studies testing progression‐free survival) or in overall survival (94.3% of studies testing overall survival). Progression‐free survival was improved in five studies (7.4% of all studies), and overall survival was improved in three studies (4.4% of all studies). Overall survival was significantly worse in two studies (3.8% of studies testing overall survival), and progression‐free survival was worse in one study (2.8% of studies testing progression‐free survival).

**FIGURE 3:**
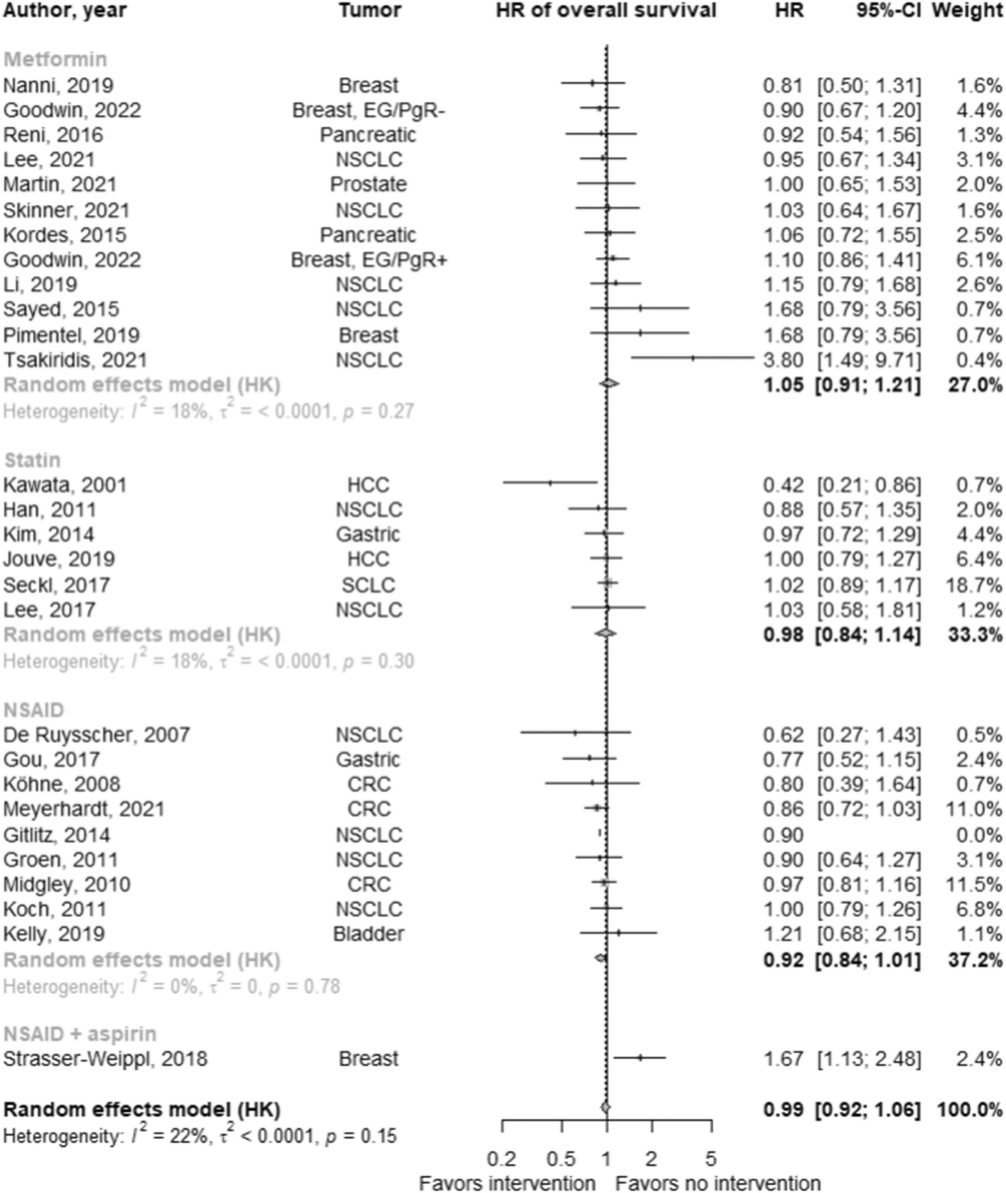
Forest plot of the effect of cardiovascular drug therapy in addition to standard of care on overall survival.

In studies reporting an overall survival hazard ratio (*n* = 32; Figure 3), the pooled hazard ratio for the effect of repurposed drugs in addition to standard of care on overall survival was 0.99 (95% CI: 0.93 to 1.06; *p* = 0.85; *I* ^*2*^: 12.2%). There were no significant differences between drug types (*χ* ^2^: 1.15; *p* = 0.56).

**FIGURE 4:**
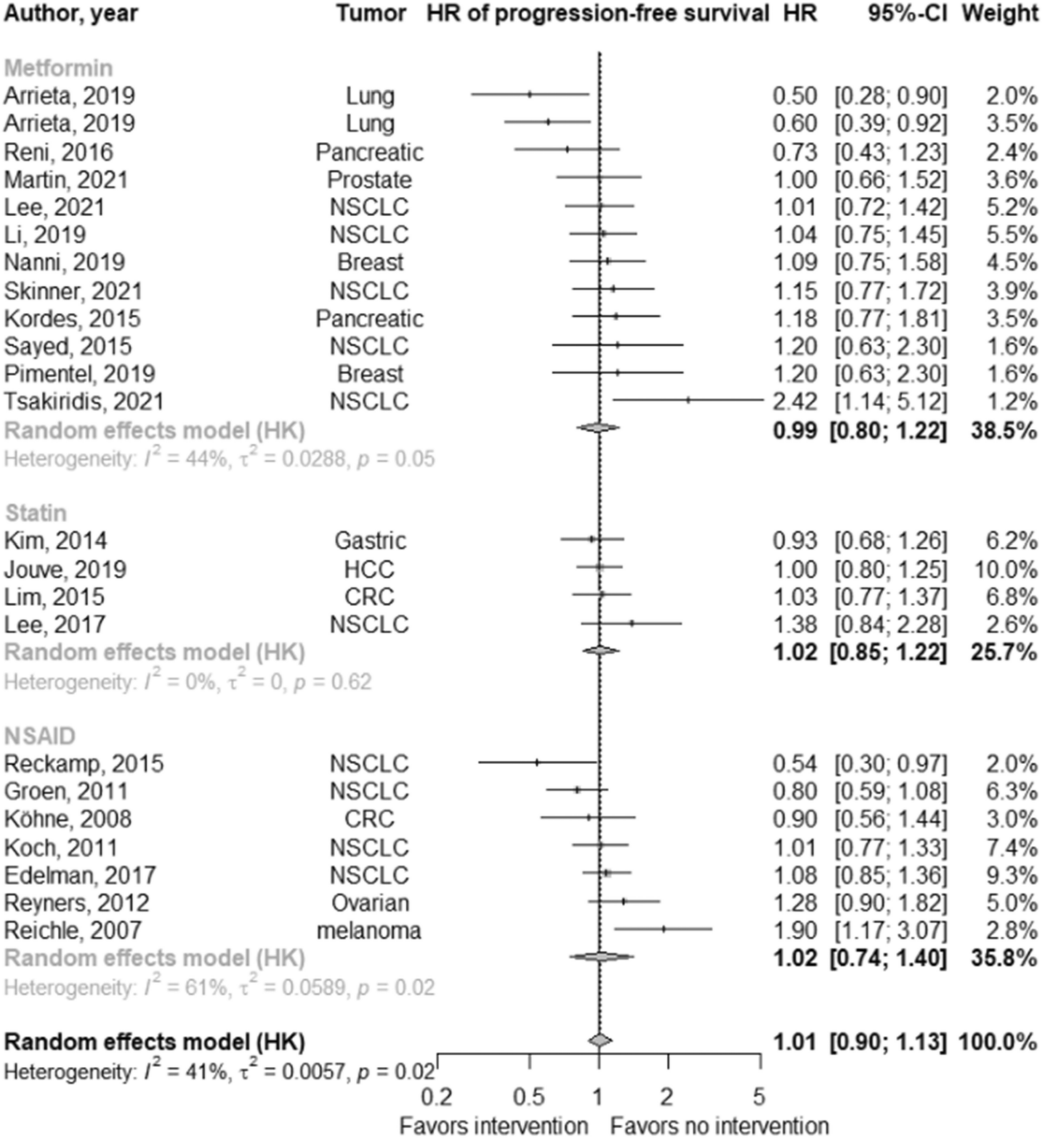
Forest plot of the effect of cardiovascular drug therapy in addition to standard of care on progression‐free survival.

In studies reporting a progression‐free survival hazard ratio (*n* = 27; Figure 4), the pooled hazard ratio for the effect of repurposed drugs in addition to standard of care on progression‐free survival was 1.02 (95% CI: 0.93 to 1.11; *p* = 0.72; *I* ^*2*^: 30.9%). There were no differences between drug types (*χ* ^2^: 0.04; *p* = 0.98).

**FIGURE 5:**
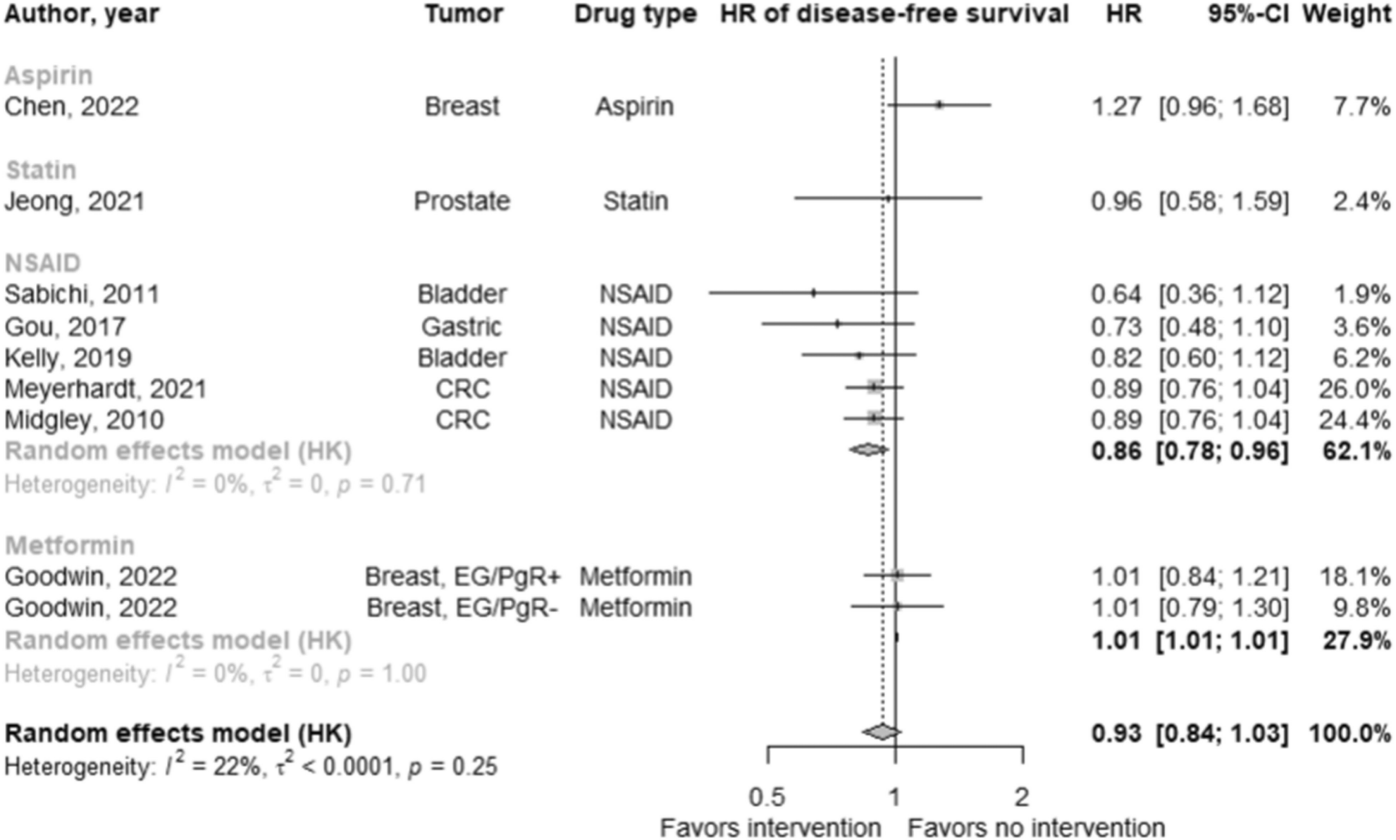
Forest plot of the effect of cardiovascular drug therapy in addition to standard of care on disease‐free survival.

In studies reporting a disease‐free survival hazard ratio (*n* = 10; Figure 5), the pooled hazard ratio for the effect of repurposed drugs in addition to standard of care on disease‐free survival was 0.94 (95% CI: 0.86 to 1.02; *p* = 0.13; *I* ^*2*^: 13.2%). There were no significant differences between drug types (*χ* ^2^: 3.70; *p* = 0.16).

**FIGURE 6 6:**
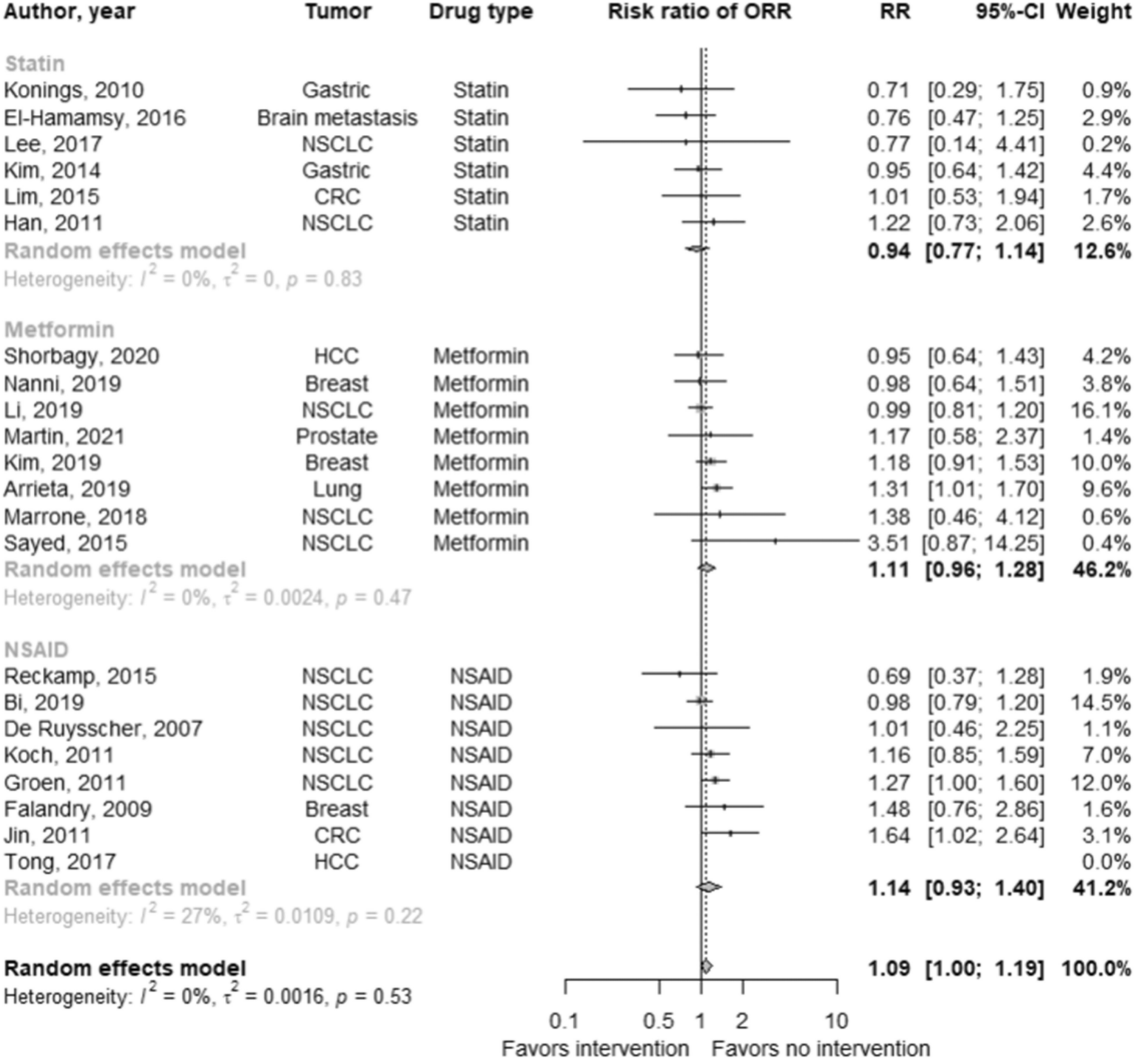
Forest plot of the effect of cardiovascular drug therapy in addition to standard of care on overall response rates.

In studies reporting overall response rates (*n* = 32; Figure 6), the pooled hazard ratio for the effect of cardiovascular drugs in addition to standard of care on overall response rates was 1.10 (95% CI: 1.02 to 1.18; *p* = 0.01; *I* ^*2*^: 18.7%). There were no differences between drug types (*χ* ^2^: 2.81; *p* = 0.24).

## 4 DISCUSSION

In this systematic review, between 2000 and 2023, most of the 67 randomized controlled trials evaluating the efficacy of cardiovascular and anti‐inflammatory drugs in treating or preventing recurrence of cancer did not demonstrate clinical benefit. In fact, three studies demonstrated worse progression‐free survival or overall survival. Only two small studies demonstrated an improvement in overall survival for advanced/metastatic non‐small‐cell lung cancer and advanced hepatocellular carcinoma. The patient population from the two small positive trials composed a small portion (0.7%, 210/28,266) of the total patient population among all randomized controlled trials.

There was a relatively equal distribution of the type of cardiovascular drug used (NSAID versus statin versus metformin) used in these clinical trials. None of the prospective randomized clinical trials that have been published in the literature have involved anti‐hypertensive medications such as ACE inhibitors and only one tested an ARB. Therefore, it is unclear if anti‐hypertensive medications may play a role in improving survival or reducing the risk of recurrence in individuals with cancer. Nearly one‐third of clinical trials involved non‐small‐cell lung cancer (19/68 = 27.9%) followed next by breast cancer (20.6%) which is consistent with the high global incidence of these malignancies.

The majority (52.9%) of studies were in the first‐line setting. As the first‐line setting is generally considered the most efficacious treatment with the greatest potential to reduce tumor burden and attempt to achieve cure, our findings suggest that these cardiovascular drugs should be avoided in studies in the first‐line setting given there was overwhelmingly no survival benefit found and potentially worsening of progression‐free survival or overall survival. Moreover, 16% (*n* = 11) of studies were conducted in the adjuvant setting where patients may already achieve cure with standard‐of‐care adjuvant therapies. It is unclear whether further trials involving these cardiovascular drugs in the adjuvant setting is worthwhile given no studies demonstrating an improvement in disease‐free survival or decrease in recurrence as per our analysis.

Despite promising pre‐clinical and observational data involving cardiovascular drugs and anti‐inflammatory drugs in treating cancer or preventing cancer recurrence, randomized controlled trial data have thus far not demonstrated the previously anticipated improvement in survival or risk reduction in recurrence. While the biological plausibility for anti‐cancer effect may be present as shown by pre‐clinical studies, it is possible that current anti‐cancer therapies such as chemotherapy and radiation are effective at disrupting specific pathways that are more influential in tumorigenesis than the pathways acted upon by cardiovascular drugs and anti‐inflammatory drugs. As such, the effect of cardiovascular and anti‐inflammatory drugs may be less significant and not translate to clinical benefit as demonstrated by this systematic review and meta‐analysis.

### 4.1 Future directions

Although the study of cardiovascular and anti‐inflammatory drugs in treating cancer does not appear to have translated to meaningful clinical value, there are growing data on the role of these drugs in prevention of cancer in high risk or healthy individuals. A systematic review and meta‐analysis of aspirin as studied mostly in healthy individuals to prevent cardiovascular outcomes evaluated colorectal cancer prevention as a secondary endpoint, with findings suggestive that high dose aspirin (as defined as 500–1200 mg per day) may reduce the risk of colon cancer development (OR 0.69, 95% CI: 0.50–0.96).^88^, ^89^ There are limited data on cancer risk reduction in healthy individuals as a primary endpoint in previously completed studies. Therefore, given the growing incidence of cancer globally, a potential avenue to explore cardiovascular and anti‐inflammatory drugs is to pivot toward prevention of cancer development in high risk and healthy individuals.

### 4.2 Limitations

This study has several limitations. Celecoxib, a COX‐2 inhibitor and NSAID that was previously equated to aspirin in cardio‐protection, was withdrawn from the market in 2004 over concerns for cardiovascular adverse effects. Several studies in our meta‐analysis included celecoxib and were halted early due to withdrawal of celecoxib. However, we have included celecoxib in our analysis as it was thought to be an anti‐inflammatory drug with cardioprotective effects at the time of each prospective trial's start date. Excluding these studies would likely have little impact on our results because of the lack of heterogeneity. Another limitation is publication bias as additional negative studies may not have been published in the literature, although this would have likely not affected our results, as our findings were largely null.

## 5 CONCLUSION

This is the first known systematic review evaluating the efficacy of cardiovascular and anti‐inflammatory drugs in treating or preventing recurrence of cancer as studied in randomized controlled clinical trials. The majority of randomized control trials evaluating the efficacy of cardiovascular drugs in oncology have not demonstrated a survival benefit or reduction in cancer recurrence. The two studies that did demonstrate survival benefit were both small studies with a total of 210 patients compared with a total patient population of 28,266 patients from all trials. Although initial pre‐clinical data as well as retrospective and cohort data for the repurposing of cardiovascular and anti‐inflammatory drugs in oncology were hopeful, the promise of improved survival or a decrease in cancer relapse has thus far failed to materialize in randomized controlled trials for individuals with cancer.

## AUTHOR CONTRIBUTIONS

**David J. Benjamin:** Conceptualization (equal); data curation (equal); formal analysis (equal); investigation (equal); methodology (equal); writing – original draft (equal); writing – review and editing (equal). **Alyson Haslam:** Conceptualization (equal); data curation (equal); formal analysis (equal); investigation (equal); validation (equal); writing – original draft (equal); writing – review and editing (equal). **Vinay Prasad:** Conceptualization (equal); formal analysis (equal); investigation (equal); supervision (lead); validation (equal); writing – original draft (equal); writing – review and editing (equal).

## CONFLICT OF INTEREST STATEMENT

V.P. has received grants from Arnold Ventures during the conduct of the study and personal fees from Johns Hopkins, MedPage, The Free Press, UnitedHealthcare, OptumRx, Patreon, YouTube, and Substack outside the submitted work. D.J.B. has the following disclosures: Consulting or Advisory Role: Seagen, Astellas, Eisai. Speakers' Bureau: Merck. Travel and Accommodations: Merck. A.H. has no disclosures.

## ETHICS STATEMENT

As our study involved publicly available data and did not involve individual patient data, this study was not submitted for institutional review board, in accordance with 45 CFR §46.102(f).

## Supporting information


Data S1:

## Data Availability

All data supporting the findings of this study are available within the paper and its supplementary information.
